# Lattice Distortion Effects on Mechanical Properties in Nb-Ti-V-Zr Refractory Medium-Entropy Alloys

**DOI:** 10.3390/ma18143356

**Published:** 2025-07-17

**Authors:** Xiaochang Xie, Ping Yang, Yuefei Jia, Yandong Jia

**Affiliations:** 1 AECC Beijing Institute of Aeronautical Materials, Beijing 100095, China; xxc127@163.com (X.X.);; 2 Institute of Materials, Shanghai University, Shanghai 200444, China; yandongjia@shu.edu.cn; 3 Zhejiang Institute of Advanced Materials, Shanghai University, Jiaxing 314100, China

**Keywords:** Lattice distortion, Medium-entropy alloys, mechanical properties

## Abstract

Medium-entropy alloys (MEAs) have attracted significant attention due to their unique structure–property relationships. In this study, we examine the effects of lattice distortion on the mechanical properties of Nb-Ti-V-Zr MEAs, focusing on two alloy series: Nb(Ti_1.5_V)_x_Zr and Nb(TiV)_x_Zr (x = 1, 2, 3, 4 and 5). Experimental results show that the Nb(TiV)_x_Zr r alloys exhibit greater atomic size mismatches and increased lattice distortion compared to the Nb(Ti_1.5_V)_x_Zr alloys, leading to higher yield strengths via enhanced solid-solution strengthening. However, excessive lattice distortion does not ensure an optimal strength–ductility balance, as the alloys with the highest distortion demonstrate limited plasticity. Thus, moderate reduction in lattice distortion proves beneficial in achieving an excellent compromise between strength and ductility. These findings offer valuable guidance for leveraging lattice distortion in the design of high-strength, high-ductility, body-centered cubic (BCC) MEAs for extreme environments.

## 1. Introduction

Medium-entropy alloys (MEAs) and high-entropy alloys (HEAs) have gained substantial attention for their outstanding mechanical properties and distinctive microstructures originating from the design concept of multiple principal elements [1,2,3]. HEAs generally consist of five or more principal elements in near-equiatomic proportions, resulting in high configurational entropy that stabilizes simple solid-solution phases. In contrast, MEAs, as a subset derived from the high-entropy alloy family, typically contain three to four principal elements, exhibiting intermediate configurational entropy between conventional alloys and HEAs. This multi-element design yields several significant advantages, encapsulated in four core effects [4]: the high-entropy effect, which promotes the formation of stable solid solution phases by increasing mixing entropy and inhibiting brittle intermetallic compound formation; the severe lattice distortion effect, attributed to atomic size discrepancies, which alters structural properties and deformation mechanisms, thereby affecting the materials’ thermodynamic and kinetic behaviors; the sluggish diffusion effect, which reduces atomic mobility and slows phase transformations, enhancing thermal stability and mechanical performance at elevated temperatures; and the cocktail effect, which arises from complex interactions among diverse alloying elements, leading to enhanced mechanical characteristics such as strength, ductility, and resistance to degradation that exceed conventional predictions [5]. Understanding these effects is crucial for advancing the design and application of HEAs across various fields, paving the way for innovative materials with tailored properties.

Recent quantitative studies have substantially advanced the understanding of lattice distortion and its critical role in shaping the mechanical properties of refractory MEAs and HEAs. For instance, Lee et al. [6,7] demonstrated that the significant lattice distortion in the NbTaTiV alloy, primarily arising from atomic size mismatch, effectively enhances both strength and ductility by altering how dislocations move and interact. The addition of zirconium Zr in the NbTaTiVZr alloy further intensifies this distortion, leading to additional mechanical property improvements. Tong et al. [8] investigated local lattice distortion in Zr- and Hf-containing multi-principal element refractory alloys, revealing distortions that surpass traditional Hume–Rothery criteria and create complex atomic-scale environments that profoundly influence deformation behavior. Similarly, Wang et al. [9] investigated the mechanical behavior of the high-entropy intermetallic alloy (CoNi)50(TiZrHf)50, emphasizing the impact of intense lattice distortion on dislocation dynamics, which enhances strength and ductility across a wide temperature range. Beyond average lattice strain, these studies collectively highlight how lattice distortion manifests as a highly heterogeneous and spatially fluctuating elastic strain field at the atomic scale, which governs dislocation morphology, mobility, and interaction mechanisms [10,11]. This heterogeneous strain landscape explains the synergistic improvement in mechanical properties observed in distorted HEAs and MEAs. Despite the critical role of lattice distortion in modulating the mechanical performance of alloys, whether increasing lattice distortion consistently results in an optimal strength–ductility combination in HEAs and MEAs remains a significant question for further inquiry.

In this study, we systematically designed and synthesized a series of Nb-Ti-V-Zr MEAs with carefully controlled compositions to tune the degree of lattice distortion. By comparing the microstructural evolution and mechanical response across these alloys, we aim to clarify the interplay between solid-solution strengthening, phase stability, and plastic deformation mechanisms. Our approach provides new insights into the optimal tuning of lattice distortion for achieving a superior strength–ductility combination in refractory complex concentrated alloys. The findings not only advance the fundamental understanding of lattice distortion effects in BCC alloys but also offer practical guidance for the design of next-generation, high-performance structural materials.

## 2. Methods

### 2.1. Alloy Fabrication

RMEA ingots with nominal compositions of Nb(TiV)_x_Zr and Nb(Ti_1.5_V)_x_Zr (where x = 1, 2, 3, 4, and 5) were fabricated by arc-melting high-purity (99.95 wt%) elemental Ti, V, Zr, and Nb under vacuum conditions. Prior to melting, the arc-melting chamber was evacuated to a high vacuum of 1 × 10^−4^ Pa. This vacuum procedure eliminated residual air, thus significantly reducing the risk of gas-related contamination. High-purity titanium was arc-melted first in the vacuum as an oxygen scavenger to further decrease the oxygen partial pressure. Each sample was flipped and melted at least five times under vacuum/argon to guarantee chemical homogeneity.

### 2.2. Material Characterization

The phase constituents were determined via X-ray diffraction (XRD) using a Rigaku D/max-2550 diffractometer (Rigaku Corporation, Tokyo, Japan) with Cu-Kα radiation operated at 40 kV. Samples for microstructural observations were prepared by mechanical polishing, followed by electropolishing at −40 °C in an electrolyte consisting of 6 vol.% perchloric acid, 34 vol.% n-butanol, and 60 vol.% methanol. The microstructure and crystallographic orientation were characterized by field emission scanning electron microscopy (SEM; ZEISS GeminiSEM 300, Carl Zeiss AG, Oberkochen, Germany), equipped with an Oxford Instruments electron backscatter diffraction (EBSD) detector (Oxford Instruments, Abingdon, UK). EBSD data were analyzed using AztecCrystal software (Version 3.0). Chemical composition analysis was performed using a JEOL EPMA-8050G electron probe microanalyzer (JEOL Ltd., Tokyo, Japan), operated at an accelerating voltage of 15 kV, a beam current of 200 nA, and a beam diameter of 100 μm.

### 2.3. Mechanical Test

Dog-bone-shaped specimens (gauge length: 2 mm, width: 1 mm, thickness: 0.5 mm) were sectioned from the as-cast ingots for uniaxial tensile testing. In total, three specimens for each alloy composition were tested to ensure reproducibility. Mechanical tests were performed at room temperature utilizing a Sans CMT-5205 microcomputer-controlled electronic testing machine (Shenzhen SANS Testing Machine Co., Ltd., Shenzhen, China), operated at a constant strain rate of 1 × 10^−3^ s^−1^ and ambient temperature.

## 3. Results and Discussions

### 3.1. Alloy Design

Based on our previous work [12,13,14,15,16], we systematically designed two series of refractory medium-entropy alloys with the general formulas Nb(TiV)_x_Zr and Nb(Ti_1.5_V)_x_Zr (where x = 1, 2, 3, 4, and 5). In these alloy systems, (TiV) denotes a 1:1 atomic ratio of Ti and V, while (Ti_1.5_V) represents a ratio of Ti:V = 1.5:1. For both alloy series, Nb and Zr are maintained at a 1:1 molar ratio, and as x increases, the fraction of (TiV) or (Ti_1.5_V) increases in the overall composition, while the Nb and Zr contents decrease proportionally to ensure the total atomic percentage sums to 100%. The specific atomic percentages for each composition are summarized in Table 1 for clarity.

**Figure 1:**
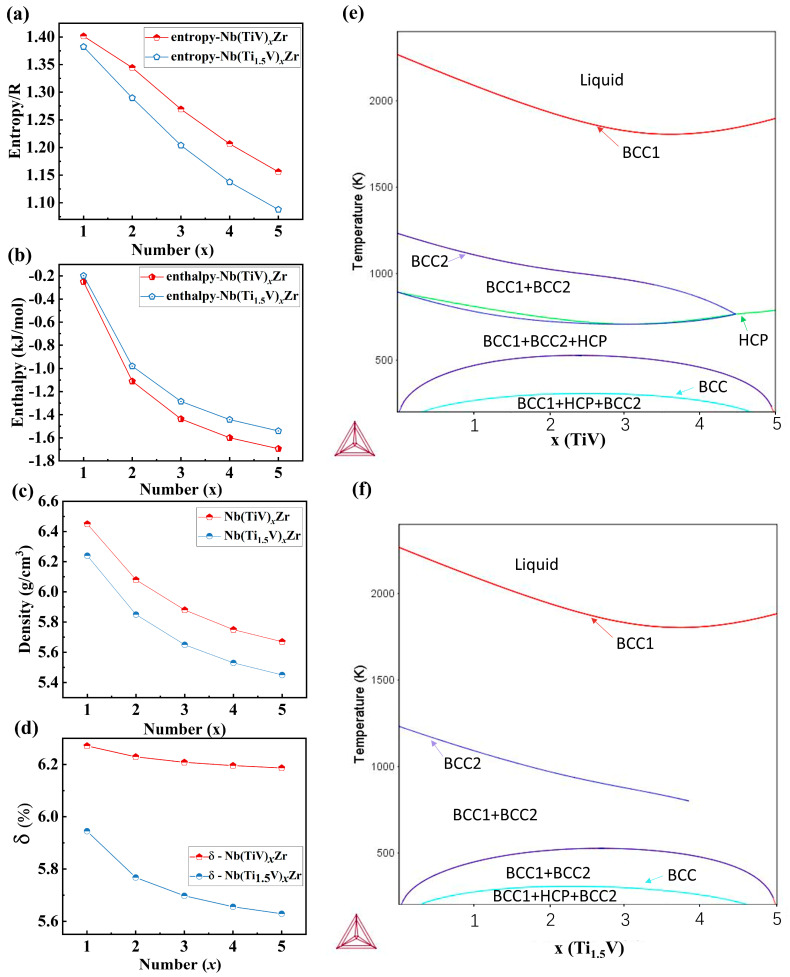
Systematic comparison of (**a**) mixing entropy, (**b**) mixing enthalpy, (**c**) density, (**d**) atomic size mismatch, and calculated phase diagrams (Thermo—Calc) for (**e**) Nb(TiV)_x_Zr and (**f**) Nb(Ti_1.5_V)_x_Zr (x = 1–5).

This design allows us to precisely modulate lattice distortion and associated properties by systematically adjusting both the (Ti/V) cluster content and the Ti/V ratio itself. Thermodynamic calculations (Figure 1a,b) demonstrate both alloy series’ yield mixing entropy values between 1.1R and 1.5R, characteristic of medium-entropy alloys. Interestingly, the Nb(TiV)_x_Zr alloys generally possess higher configurational entropy but exhibit a more negative mixing enthalpy compared to the Nb(Ti_1.5_V)_x_Zr alloys at the same x. Density measurements (Figure 1c) show both alloy series have values ranging from 5.4 to 6.5 g/cm^3^, with Nb(TiV)_x_Zr alloys displaying higher density than Nb(Ti_1.5_V)_x_Zr alloys.

Most critically, we quantified atomic size mismatch (*δ*) using the following established formula [17]:

(1) δ=∑i=1nci1−rir¯2

(2) $$\bar{r}=\overset{n}{\underset{i=1}{\sum }}c_{i}r_{i}$$

where $\bar{r\;}\;$ represents the average atomic radius, and *r_i_* is the radius of the *i*-th element. Our analysis revealed that Nb(TiV)_x_Zr alloys exhibit significantly greater *δ* compared to Nb(Ti_1.5_V)_x_Zr alloys (Figure 1d), indicating higher lattice distortion [18].

Furthermore, equilibrium phase diagrams for both alloy series were calculated using the CALPHAD (Calculation of Phase Diagrams) approach, as shown in Figure 1e,f. The CALPHAD results indicate that both Nb(TiV)_x_Zr and Nb(Ti_1.5_V)_x_Zr systems feature extensive single-phase BCC regions across the studied compositional space. Notably, as the value of x increases, the BCC single-phase stability range widens in both alloys, suggesting enhanced phase stability with increased (TiV) or (Ti_1.5_V) content.

For Nb(TiV)_x_Zr (Figure 1e), the phase diagram reveals the appearance of an HCP (hexagonal close-packed) phase at lower temperatures and higher x values, leading to a multiphase region (BCC + BCC2 + HCP). In comparison, the Nb(Ti_1_._5_V)_x_Zr system (Figure 1f) maintains a predominantly BCC phase field throughout most of its composition range, with only a limited two-phase BCC region at lower temperatures.

### 3.2. Microstructure Analysis

**Figure 2:**
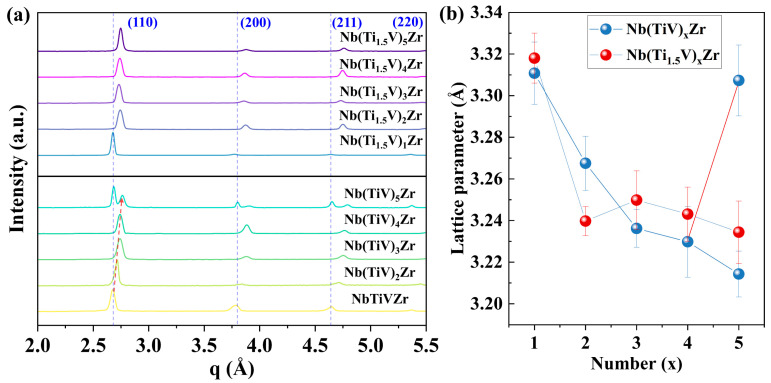
Crystal information of Nb(TiV)_x_Zr and Nb(Ti_1.5_V)_x_Zr (with x = 1, 2, 3, 4, and 5). (**a**) XRD patterns; (**b**) lattice parameters.

Figure 2a presents the XRD patterns of Nb(TiV)_x_Zr and Nb(Ti_1.5_V)_x_Zr samples, with x values of 1, 2, 3, 4, and 5. The Nb(Ti_1.5_V)_x_Zr alloys consistently exhibit a BCC structure across all compositions. In contrast, the Nb(TiV)_x_Zr alloys maintain a BCC structure for x = 1, 2, 3, and 4, while the sample with x = 5 demonstrates phase separation into two distinct BCC phases, potentially correlating with the more negative mixing enthalpy of Nb(TiV)_5_Zr. Additionally, for Nb(TiV)_x_Zr alloys (x = 1 to 4), a notable rightward shift in primary peak positions indicates a reduction in interplanar spacing. This trend is attributed to changes in atomic interactions and lattice parameters caused by varying Ti and V concentrations.

Notably, all samples were synthesized under high vacuum conditions, effectively minimizing the influence of interstitial impurities such as oxygen and nitrogen, and ensuring that the observed phase behavior and lattice characteristics are intrinsic to the alloy compositions.

The calculated lattice parameters for both systems, as shown in Figure 2b, reveal distinct trends. For Nb(TiV)_x_Zr (x = 1, 2, 3, and 4), there is a continuous decrease in lattice parameters with increasing x, culminating in the formation of two distinct BCC cells at x = 5. This behavior suggests a critical threshold for solute accommodation, leading to phase separation as a mechanism for relieving internal stresses. Conversely, Nb(Ti_1.5_V)_x_Zr samples maintain a stable BCC structure across all compositions, with lattice parameters initially decreasing with increasing x, followed by an increase and a subsequent decrease, indicating a complex interplay between composition and structural stability.

**Figure 3:**
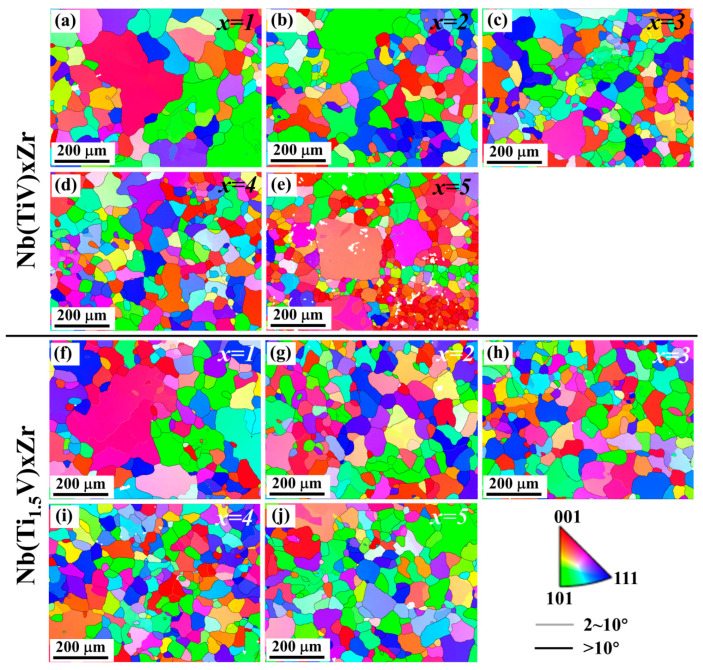
Electron backscatter diffraction (EBSD) inverse pole figure (IPF) maps of (**a**–**e**) Nb(TiV)_x_Zr and (**f**–**j**) Nb(Ti_1.5_V)_x_Zr (for x = 1, 2, 3, 4, and 5).

**Figure 4:**
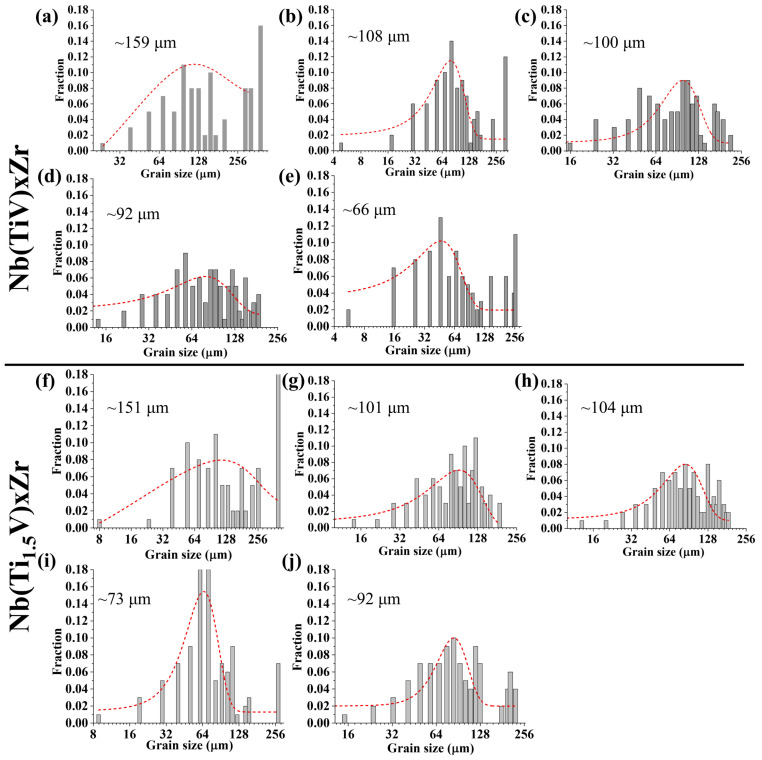
Grian size distribution of (**a**–**e**) Nb(TiV)_x_Zr and (**f**–**j**) Nb(Ti_1.5_V)_x_Zr (for x = 1, 2, 3, 4, and 5), with the average grain size and fitted curves (in red) shown in the figure.

Figure 3 presents the EBSD inverse pole figure (IPF) maps for Nb(TiV)_x_Zr and Nb(Ti_1.5_V)_x_Zr alloys, where x = 1, 2, 3, 4, and 5. The microstructures of Nb(TiV)_x_Zr (Figure 3a–e) reveal distinct characteristics depending on the value of x. The NbTiVZr alloy (x = 1) predominantly exhibits an equiaxed grain structure, with a significant presence of larger grains exceeding 200 μm and an average grain size of approximately 159 μm (Figure 4a). In the case of Nb(TiV)_2_Zr (x = 2), the microstructure is similar to that of NbTiVZr, maintaining a uniform grain orientation while still containing a few larger grains and an average grain size of about 108 μm (Figure 4b). The grain morphology continues in Nb(TiV)_3_Zr and Nb(TiV)_4_Zr (x = 3 and 4), where the grains become finer and more uniform, with average sizes of approximately 100 μm (Figure 4c) and 92 μm (Figure 4d).

**Figure 5:**
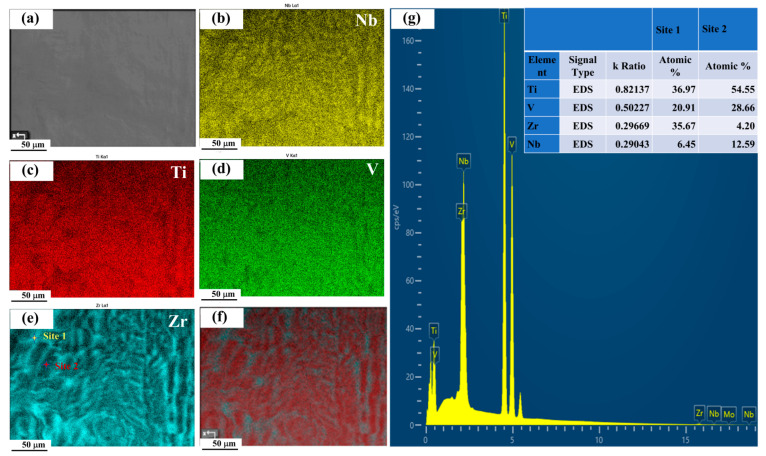
EDS analysis of the Nb(TiV)_5_Zr alloy: (**a**) corresponding BSE image; (**b**–**e**) elemental distribution maps of Nb, Ti, V, and Zr; (**f**) overlap map of Ti and Zr; (**g**) EDS spectra and atomic percentages for the two selected regions.

Nb(TiV)_5_Zr (x = 5) shows a notable increase in unindexed regions alongside the presence of several large grains, with a reduced average grain size of around 66 μm (Figure 4e). The instability of grain structure in this alloy could suggest competition between grain growth and phase stability during processing. Additionally, Energy-Dispersive X-ray Spectroscopy (EDS) analysis (Figure 5a–g) reveals notable compositional heterogeneity across the sample. The elemental mapping (Figure 5b–e) indicates that Zr-rich regions are correspondingly depleted in Ti, V, and Nb. Quantitatively, the Zr-enriched area (Site 1) has an approximate atomic composition of Ti 36.97%, V 20.91%, Zr 35.67%, and Nb 6.45%, while the Ti-rich area (Site 2) contains about Ti 54.55%, V 28.66%, Zr 4.20%, and Nb 12.59% (Figure 5g). These compositional variations further confirm the existence of dual BCC phases, as previously identified (see Figure 2a).

For the Nb(Ti_1.5_V)_x_Zr series (where x = 1, 2, 3, 4, and 5), the microstructural features are illustrated in Figure 3f–j. Similarly to NbTiVZr, NbTi_1.5_VZr (x = 1) displays a limited number of large grains, with an average grain size of approximately 151 μm (Figure 4f). In contrast, the alloys Nb(Ti_1.5_V)_x_Zr (where x = 2, 3, 4, and 5) predominantly exhibit an equiaxed grain morphology across the series. The average grain sizes are measured as 101 μm (Figure 4g), 104 μm (Figure 4h), 73 μm (Figure 4i), and 92 μm (Figure 4j), respectively. A slight reduction in grain size is observed at x = 4. Overall, the systematic changes in grain morphology and size across the Nb(TiV)_x_Zr and Nb(Ti_1.5_V)_x_Zr series underscore the influence of alloy composition on microstructural development.

**Figure 6:**
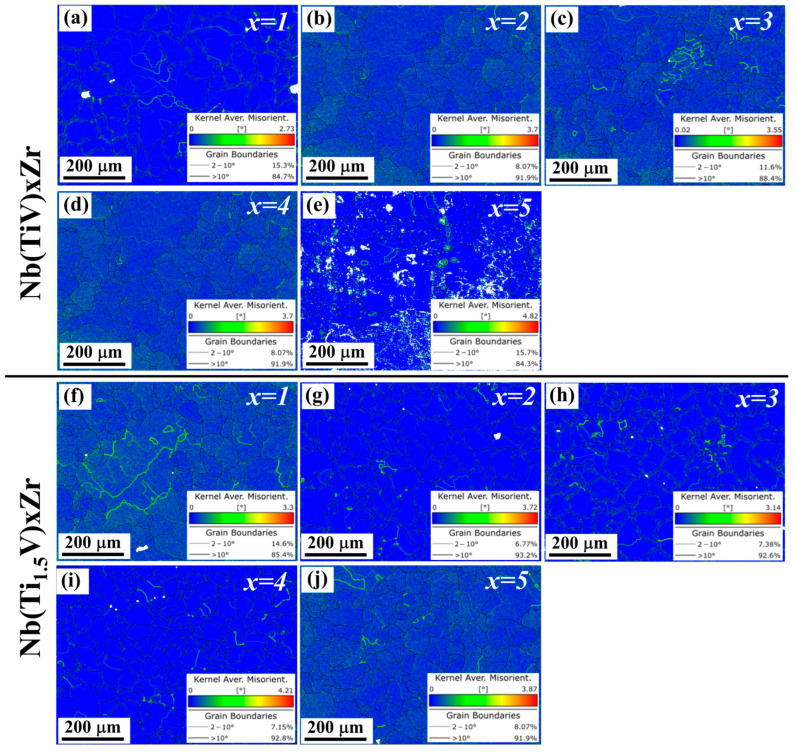
Kernel average misorientation (KAM) maps of (**a**–**e**) Nb(TiV)_x_Zr and (**f**–**j**) Nb(Ti_1.5_V)_x_Zr (for x = 1, 2, 3, 4, and 5).

Figure 6 delineates the heterogeneous distribution of kernel average misorientation (KAM) in as-cast Nb(TiV)_x_Zr and Nb(Ti_1.5_V)_x_Zr alloys (x = 1–5), revealing composition-dependent strain localization patterns. For the Nb(TiV)_x_Zr series, KAM values exhibit progressive intensification with increasing x; the x = 1 alloy displays minimal misorientation concentrated at sporadic subgrain boundaries, characteristic of near-homogeneous solidification. Intermediate compositions (x = 2–4) develop intragranular KAM bands, aligning with slip system activation during eutectic phase growth. At x = 5, pronounced KAM escalation emerges at dual-phase interfaces, demonstrating interfacial strain accumulation from lattice mismatch.

In the Ti-enriched Nb(Ti_1.5_V)_x_Zr system, KAM distributions exhibit contrasting behavior; both x = 1 and x = 5 alloys maintain uniform low-angle misorientation across grain interiors, consistent with single-phase stability. The x = 2–4 variants show bimodal KAM partitioning within grain boundaries. Crucially, all specimens exhibit KAM magnitudes below θ = 2.0°, confirming limited residual strain in these MEAs.

### 3.3. Mechanical Properties

**Figure 7:**
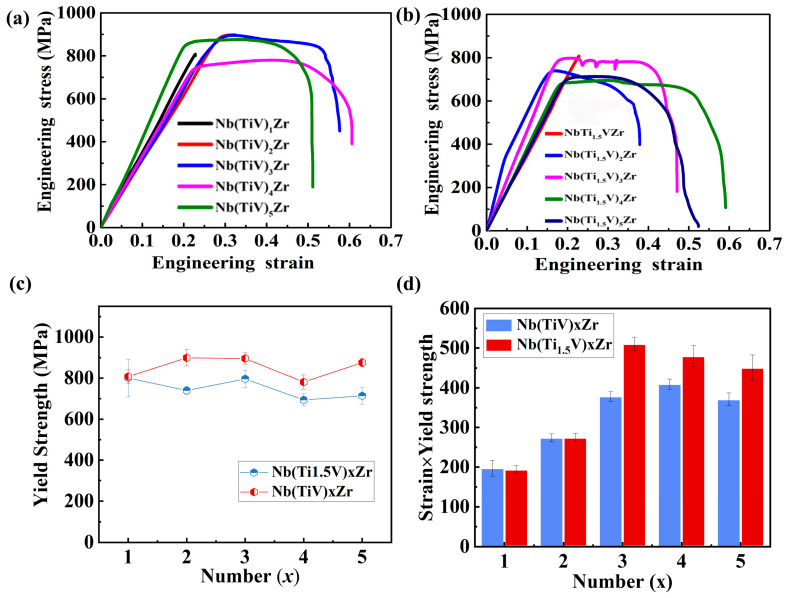
Mechanical properties of Nb(TiV)_x_Zr and Nb(Ti_1.5_V)_x_Zr (for x = 1, 2, 3, 4, and 5). (**a**) Tensile engineering stress–strain curves of Nb(TiV)_x_Zr; (**b**) tensile engineering stress–strain curves of Nb(Ti_1.5_V)_x_Zr, with an image of the fractured tensile specimen on the right; (**c**) comparison of yield strength; (**d**) comparison of strength × strain.

The tensile engineering stress–strain curves for Nb(TiV)_x_Zr and Nb(Ti_1.5_V)_x_Zr alloys (where x = 1, 2, 3, 4, and 5) are illustrated in Figure 7a,b. In Figure 7a, NbTiVZr exhibits a yield strength of approximately 800 MPa, without demonstrating any tensile plasticity. Nb(TiV)_2_Zr shows a slightly higher yield strength of about 900 MPa, accompanied by limited tensile plasticity. Interestingly, Nb(TiV)_3_Zr displays a yield strength comparable to that of Nb(TiV)_2_Zr but outperforms it in terms of tensile plasticity. Conversely, Nb(TiV)_4_Zr presents the lowest yield strength at around 730 MPa, yet it possesses the highest fracture strain among all Nb(TiV)_x_Zr alloys. Nb(TiV)_5_Zr reaches a yield strength of approximately 850 MPa, exhibiting moderate tensile plasticity.

In Figure 7b, the NbTi_1.5_VZr alloy is characterized by its lower strength but remarkable tensile plasticity. Nb(Ti_1.5_V)_2_Zr has a yield strength of about 720 MPa, again showing limited tensile plasticity. The yield strength of Nb(Ti_1.5_V)_3_Zr rises to approximately 800 MPa, which is accompanied by improved tensile plasticity. Notably, Nb(Ti_1.5_V)_4_Zr achieves a yield strength of around 700 MPa and demonstrates optimal fracture strain. Nb(Ti_1.5_V)_5_Zr has a yield strength of approximately 710 MPa; however, its plasticity is significantly lower compared to Nb(Ti_1.5_V)_4_Zr.

Figure 7c compares the yield strengths of Nb(TiV)_x_Zr and Nb Nb(Ti_1.5_V)_x_Zr alloys (where x = 1, 2, 3, 4, 5), revealing that the Nb(TiV)_x_Zr series consistently exhibits higher yield strengths than the Nb(Ti_1.5_V)_x_Zr alloys. This can be attributed to the greater lattice distortion in the Nb(TiV)_x_Zr series (*δ* > 6.2%), compared to the (Ti_1.5_V)_x_Zr alloys (*δ* < 5.96%).

Analysis of the strength–ductility product (Figure 7d) further shows that the Nb(TiV)_3_Zr alloy achieves the most favorable balance between strength and ductility. Interestingly, this result challenges the straightforward assumption that higher lattice distortion directly leads to better mechanical properties. In fact, the equiatomic NbTiVZr alloy—with the highest measured lattice distortion (*δ* > 6.28%)—exhibits the lowest strength and ductility in the series. In contrast, the Nb(Ti_1.5_V)_3_Zr alloy, which has a moderate lattice distortion (*δ* ≈ 5.7%), achieves the best combination of strength and ductility.

This phenomenon can be explained by considering the underlying deformation mechanisms in HEAs and MEAs. Increased lattice distortion generates highly heterogeneous local strain fields, which serve as strong barriers to dislocation movement and thus enhance solid solution strengthening [19]. As discussed in our previous work [13,14], moderate lattice distortion facilitates the activation of multiple slip systems and promotes dislocation multiplication through mechanisms such as enhanced Frank–Read sources and jog formation [11,13]. This improves strain-hardening capacity and helps maintain an optimal balance between strength and ductility [13,14].

However, excessive lattice distortion can severely impede dislocation mobility and increase local lattice friction stress. This leads to an accumulation of immobile dislocations and localized strain concentrations, which encourage early strain localization and reduce ductility. Furthermore, significant lattice distortion can increase the energy barrier for screw dislocation movement and synchronize the velocities of screw and edge dislocations, heightening dislocation–dislocation interactions and further hindering plastic flow [20]. This understanding is consistent with recent studies on dislocation dynamics and plasticity in HEAs [10] and explains why excessive lattice distortion in our alloys leads to reduced ductility rather than continued mechanical improvement.

The yield strength (*σ_y_*) of these alloys primarily results from the combined effects of solid-solution strengthening (*σ_ss_*) and grain boundary strengthening (*σ_gb_*), with dislocation-related contributions being negligible due to the characteristically low dislocation densities revealed by KAM analysis (Figure 6). Thus, the overall yield strength can be approximated by

(3) $$\;\sigma_{y}\approx \sigma_{ss}+\sigma_{gb}$$

**Figure 8:**
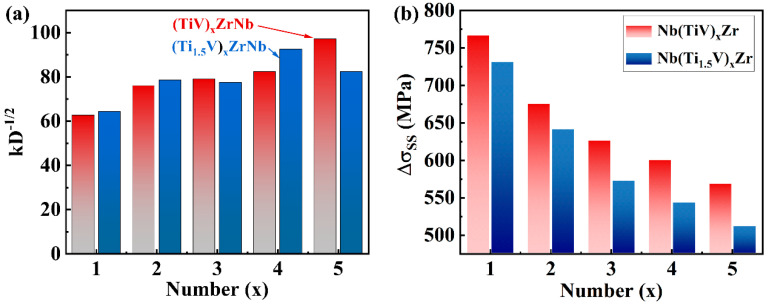
Strengthening analysis: (**a**) grain boundary strengthening increment, kD^−1/2^; (**b**) solid-solution strengthening (Δ*σ_SS_*).

*σ_gb_* in these alloys can be quantitatively described by the following classical Hall–Petch equation:

(4) $$\sigma_{gb}=\sigma_{0}+kD^{-\frac{1}{2}}$$

where *σ*_0_ denotes the lattice friction stress [13]. The parameter k represents the Hall–Petch slope, measured here as 7.9 MPa·$\sqrt{\mathrm{mm}}$ [21], and *D* corresponds to the average grain size in millimeters, as presented in Figure 4. The calculated grain size-dependent strengthening increment ranges from 40 to 90 MPa (Figure 8a), indicating a relatively modest contribution to the overall strength.

Consequently, the high strength of Nb-Ti-V-Zr MEAs primarily arises from lattice distortion, which contributes to strengthening through solid solution effects [6,22]. Senkov et al. [23] introduced a solid-solution strengthening model specifically tailored for BCC HEAs, extending the Labush model [24]. This framework assesses the size and modulus mismatches between different atomic pairs, aggregating these factors according to the alloy’s crystal structure to evaluate overall lattice distortion and modulus mismatch.

The solid solution strengthening can be mathematically represented as follows:

(5) $$\sigma_{ss}={\left(\sum_{i=1}^{n}\Delta \sigma_{i}^{\frac{3}{2}}\right)}^{\frac{2}{3}}$$

In this equation, Δ*σ_i_* quantifies the strengthening contribution from the *i*-th element, given by

(6) $$\Delta \sigma_{i}=\gamma G\omega_{i}^{\frac{4}{3}}c_{i}^{\frac{2}{3}}\;\;\;\;\;\;\;$$

where *γ* is a dimensionless parameter, empirically determined to be 0.04 based on analyses of various HEAs [23,24], *c_i_* denotes the atomic percentage of the alloying element, and *G* represents the shear modulus of the alloy. The expression for *ω_i_* is defined as

(7) $$\;\;\;\;\;\;\;\;\;\;\;\;\omega_{i}=\sqrt{\delta_{Gi}^{2}+\alpha^{2}\delta_{ri}^{2}}$$

In this context, *α* indicates the contributions of different types of dislocations during deformation, with higher values suggesting a greater impact from screw dislocations over edge dislocations [20]. For the studied single-phase BCC alloys, α is set to 9.

The shear modulus mismatch *δ_Gi_* is described by

(8) $$\delta_{Gi}=\varphi \sum_{i=1,\;i\neq j}^{n}c_{j}\delta_{Gij}$$

(9) $$\;\;\;\delta_{G_{ij}}=\frac{2\left(G_{i}-G_{j}\right)}{\left(G_{i}+G_{j}\right)}$$

Here, *G_ij_* represents the shear modulus of pure metal *j* as sourced from the literature [12]. Additionally, *ϕ* is a parameter that varies with crystal structure; for the BCC structure examined, it equals 9/8. The atomic size-induced mismatch, *δ_ri_* is expressed as

(10) $$\delta_{ri}=\varphi \sum_{i=1,i\neq j}^{n}c_{j}\delta_{rij}$$

(11) $$\;\;\delta_{rij}=\frac{2\left(r_{i}-r_{j}\right)}{\left(r_{i}+r_{j}\right)}$$

Results from calculations of solid-solution strengthening in Nb(TiV)_x_Zr and Nb(Ti_1.5_V)_x_Zr alloys (for x = 1, 2, 3, 4, and 5) are illustrated in Figure 8b. The findings reveal that the solid-solution strengthening effect diminishes with increasing x, which corresponds with the observed reduction in lattice distortion as x increases. Notably, Nb(TiV)_x_Zr alloys demonstrate a greater solid-solution strengthening effect compared to Nb(Ti_1.5_V)_x_Zr alloys. This is linked to the higher levels of lattice distortion in the Nb(TiV)_x_Zr alloys, which correlates with tensile results showing enhanced strength.

## 4. Conclusions

In summary, this study investigated the correlation between lattice distortion, induced by atomic size mismatch, and mechanical properties in the Nb(TiV)_x_Zr and Nb(Ti_1.5_V)_x_Zr alloy series (x = 1, 2, 3, 4, 5). The results demonstrate that Nb(TiV)_x_Zr alloys exhibit significantly greater lattice distortion (*δ* > 6.2%) compared to the Nb(Ti_1.5_V)_x_Zr series (*δ* < 5.96%). While enhanced lattice distortion contributes to increased yield strength through solid-solution strengthening, excessive distortion does not necessarily ensure superior strength–ductility synergy. The equiatomic NbTiVZr alloy, despite possessing the highest lattice distortion, displays inferior mechanical properties with the lowest strength and ductility among all compositions examined. Conversely, the Nb(Ti_1.5_V)_3_Zr alloy (*δ* ≈ 5.7%) achieves the most favorable strength–ductility combination. The findings indicate that in systems with limited plasticity, moderate lattice distortion levels can facilitate optimal strength–ductility combinations. These insights provide valuable guidance for designing next-generation, high-strength, ductile BCC refractory MEAs and emphasize the importance of precisely controlling lattice distortion for property optimization.

## Figures and Tables

**Table 1:** Chemical composition (at.%) and density (g/cm^3^) of the prepared alloys.

Composition	Density	Atomic Percent (Calc.)				Atomic Percent (Meas.)				
		Ti	V	Zr	Nb	Nb	Ti	V	Zr	Hf
(Ti_1.5_V)_1_ZrNb	6.24	33.4	22.2	22.2	22.2	23.41	32.17	21.65	22.31	0.46
(Ti_1.5_V)_2_ZrNb	6.08	42.9	28.5	14.3	14.3	13.85	43.07	28.79	14.06	0.23
(Ti_1.5_V)_3_ZrNb	5.85	47.4	31.6	10.5	10.5	8.25	48.17	35.22	8.19	0.17
(Ti_1.5_V)_4_ZrNb	5.53	50	33.4	8.3	8.3	7.44	53.12	32.47	6.84	0.13
(Ti_1.5_V)_5_ZrNb	5.45	51.7	34.5	6.9	6.9	26.42	26.35	23.5	23.18	0.55
TiVZrNb	6.45	25	25	25	25	16.65	35.42	31.58	16.01	0.34
(TiV)_2_ZrNb	6.12	33.3	33.3	16.7	16.7	12.5	35.88	39.14	12.23	0.25
(TiV)_3_ZrNb	5.88	37.5	37.5	12.5	12.5	10.05	41.82	38.03	9.89	0.21
(TiV)_4_ZrNb	5.75	40	40	10	10	7.74	43.62	41.34	7.16	0.14
(TiV)_5_ZrNb	5.67	41.7	41.7	8.3	8.3	23.41	32.17	21.65	22.31	0.46

Calculated composition (calc.); measured composition (meas.).

## Data Availability

The original contributions presented in the study are included in the article; further inquiries can be directed to the corresponding authors.
